# St. George's Hospital

**Published:** 1830-11

**Authors:** 


### HOSPITAL REPORTS.

### ST. GEORGE'S HOSPITAL.

### *Cases of Fistula Perinei.**

Case I. William Lord, ætatis sixty-eight, admitted June 2, 1830, under the care of Mr. Babington.

Has numerous fistulous openings in the perineum and scrotum, especially on the right side; another fistulous opening over the right abdominal ring: the various openings communicate more or less directly with each other, undermining the integument, which is blue, unhealthy, and excoriated. The urine escapes through all. No instrument whatever can be passed into the bladder.

By the patient's own account, no urine has passed through the meatus for nearly ten years, during which period he has had the various fistulæ in perineo. Has had stricture for twenty years.

On the 5th several sinuses were laid into one, in order to afford a more ready exit to the urine, and prevent its spreading towards the pubic openings. On the 11th he was seized with a violent rigor, speedily followed by another: the integuments of the perineum looked red, and the wounds were sloughy. Reaction to a slight extent succeeded, but there was no pain in the loins to demonstrate mischief in the kidneys, no evidence of erysipelas. Æther mixture and camphor were given internally; poultices, and the recumbent position, were the external agents.

* Medical Gazette.

On the 14th the subcutaneous cellular membrane of the perineum and posterior part of the scrotum were evidently infiltrated with urine; the former was inclined to slough. The patient had been sick in the morning, and was very low, but he had experienced no return of the rigors.—Catap. Fermenti. Haust. Cinch. c. Tinct. Op. ♏v. t. d.

On the 15th he appeared to be dying, and displayed all the symptoms so characteristic of the prostration produced by urinous effusion. The countenance was sunken, sallow, hectic; the mind wandering; the pulse feeble, rapid, intermittent; the tongue dry, glazed, and red; the bowels disposed to diarrhœa. The cellular membrane of the perineum was sloughing, the infiltration extending to the dependent parts of the scrotum, and the skin in that direction assuming the peculiar erythematous tint.—Lotio Acid. Muriat. c. gtt. iij. ad Aquæ ℥j. Vin. rubri ℥iv. Sp. Vin. Gall. ℥iv. quotidie. Cataplasmata.

We believe that some incisions were made by Mr. Babington, but at all events the patient made a rally, and for two or three days did surprisingly well. The sloughing stopped, and the urine was freely discharged from the wound, for nearly the whole were now merged into one.

In the afternoon of the 17th he again had a rigor, and from this time to the 20th he went on with little alteration, being perhaps rather worse than better. He was very low, with feeble, intermitting pulse; dry, glazed tongue; and great despondency of mind. The state of the parts in the perineum varied little, being sloughy, with red, inflamed skin around.

In the evening of the 20th he died.

*Sectio Cadaveris.* Body emaciated.

Integuments of the whole perineum removed by ulceration and sloughing. The latter had not passed below the subcutaneous cellular membrane, but sinuses ran more deeply in various directions. One passed round the penis on the left, and debouched into an ulcerated opening of some size on the right side of the pubes. The anterior portion of the urethra ended about the root of the penis in a perfect cul-de-sac. Beyond this extended, for an inch and a half, a solid callous mass, in which corpora cavernosa and spongiosa were blended, and no canal could be discovered. About this spot were numerous sinuses.

The bladder was very capacious, not much thickened, and free from ulceration. The membranous part of the urethra was dilated, and about two inches from the caput gallinaginis it ended in two large apertures, leading to the perineum, near the arch of the pubes. These openings, originally formed by ulceration, passed through the upper part of the urethra.

Kidneys small and puckered; left studded with small vesicles, and presenting one or two portions not unlike the medullary degeneration; right displaying still more of this appearance, and showing some approaches to the scrofulous tubercle.

Liver very soft, and of nutmeg colour.

Thorax: Heart flabby, and rather large.

Case II. Robert White, ætatis fifty-nine, a country labourer, admitted July 14, 1830, under the care of Mr. Babington.

Swelling, of the size of a duck's egg, in the centre of the inferior part of the scrotum; no tumefaction of the perineum. Symptoms rather low and typhoid, but not to any extent: appearance unhealthy.

By his own statement, has had stricture for eighteen years, and bougies have been often introduced. Present symptoms have been coming on for a fortnight or three weeks.

The tumor was punctured, and the contents of a sloughy, urinous abscess discharged, but a good deal of induration remained at its sides. A small catheter, without a stilette, was introduced with little difficulty into the bladder, but it seemed to pass through a gristly false passage, and some pus escaped through it before the water issued. The quantity of the latter was then and afterwards remarkably small. The catheter was ordered to be kept in the bladder at night, and withdrawn during the day.—Catapl. lini.

15th. Symptoms rather more typhoid; no sickness nor rigor; sloughing of cellular membrane of scrotum.—Mist. Camph. c. Ammon. Carb. gr. v.; Liq. Op. Seq. ♏vj. 6tis horis.

16th. Feels lower; pulse feeble, not very frequent; tongue dry, and rather brown in the centre; night delirium. Wound much the same. Arrowroot. Vin. rub. ℥viij. Cerevis. fortis ℥viij.

Wound injected with solution of chloride of soda, filled with carrot, and covered with a poultice.

Vespere. Prostration increasing.—H. Cinch. c. Acid. Sulph. Dil. ♏xv. 6tis horis. Om. Mist. Camph.

17th. Diarrhœa, wound cleaning.

21st. Has continued with little alteration, having never fairly rallied from his feeble state. Has a tendency to low delirium, and is constantly crying; pulse feeble; bowels disposed to diarrhœa; urine at times contains pus. Is troubled with nausea; no rigor. The sloughing of the scrotum has entirely ceased, and the wound is becoming clean. The symptoms of sinking rapidly advanced, and on the 23d he died. The instrument could be passed from the penis out through the wound.

*Sectio Cadaveris.* Body not much emaciated.

Abdomen: Kidneys large, flabby, mottled in colour, with small yellowish spots dispersed through them. When cut open, they looked like Castile soap, but were paler. Parts were minutely injected with blood-vessels; parts showed a deposit, apparently of partially organised lymph. The distinction between cortical and tubular structure was obscured. The whole was, no doubt, a specimen of chronic inflammation of the kidney. The inner membrane of the ureters and pelvis was inflamed; in one it was covered with lymph. The ureters were not dilated.

The bladder was small, contracted, thickened; its mucous membrane in a state of chronic inflammation, but not ulcerated. The urethra, from the caput-gallinaginis to the urethral end of the membranous portion, was extremely dilated. Beyond this, the proper canal of the urethra was entirely obstructed; but a false passage extended from the farther confine of the obstruction, by the side of the natural channel, and opened into the bladder beneath the caput-gallinaginis. The obstructed part of the urethra was solid, and of gristly hardness. The tunica vaginalis of the testes was mixed up in the indurated walls of the sloughy abscess in the scrotum. This, of course, communicated both with the urethra contained in the penis and the false passage leading to the bladder. It formed a sort of entrepôt between them.

Thorax: Nothing particular. Cranium, not examined.

---

The ordinary reports of cases are often so imperfect, that it is scarcely fair to censure, upon their evidence alone, the practice that has been adopted: if, however, the report of the above cases is correct, we certainly think that an operation might have been performed in them with some chance of benefit, which it does not appear was attempted. In order to obtain a third opinion, either in refutation of our own, or in confirmation of it, we requested a hospital surgeon, whom we had seen operate with success in cases similar to the above, to give us the results of his experience upon the operation of dividing strictures of the urethra. The following is the communication with which he has favored us.

" The operation of dividing a stricture in the urethra, *situated in the usual seat of stricture,* viz. at the junction of the bulb with the membranous portion, may be performed with great quickness in the following manner:

Introduce a middling-sized staff into the urethra, till it reaches the stricture; then, feeling the end of the instrument from the perineum, cut down upon it, the incision made through the integuments being one inch and a half in length, and parallel and close to the raphe, the incision into the urethra being half an inch in length. Withdraw the staff, after having exactly seen and felt where it points when held in the usual position towards the bladder; then thrust the scalpel forward in the line of the contracted and indurated urethra, one half or three fourths of an inch. Next, introduce along the urethra a middling-sized catheter, and try if it will pass into the bladder: it probably will; but if it be still obstructed, withdraw it a little, and lengthen the incision that has been already made, if the part beyond be firm and cartilaginous; or from the point where the previous incision began, thrust the knife half an inch forwards, with the edge directed upwards, instead of transversely, as before: the catheter will afterwards certainly find a ready passage onward to the bladder.

The urethra *beyond the stricture* is sure of being opened by incisions made in this manner, provided the operator possess a correct anatomical knowledge of the parts, as that portion of the canal is always capacious in such cases, having been dilated by the long-continued pressure of the urine. Those who fail in performing this operation, fail from want of boldness, losing time in endeavouring to find a channel through the stricture by means of a probe or small catheter, after the urethra is cut into, which may serve as a guide in the division of the stricture. Such a guide is not needed by one who relies upon sufficient anatomical knowledge, nor should be thought of by the operator, if, as is likely, he has already found the urethra impervious to the smallest instruments.

The operation is concluded by fixing the silver catheter in the bladder. The silver catheter had better be removed in forty-eight hours, and an elastic one substituted, which is to be changed for another every day subsequently, till the wound has cicatrized; at which time a urethra is left, which has indeed some tendency to contract, but which requires only, for the prevention of this consequence, the occasional introduction of a bougie.

The operation which has been described is applicable, 1st, to stricture complicated with rupture of the urethra; 2d, to certain cases of stricture complicated with fistula perinæi; 3d, to certain cases of simple permanent stricture.

I. Rupture of the urethra, in the ordinary situation in which that lesion occurs, is characterized by sudden and considerable swelling of the penis, scrotum, and perineum, followed quickly by discoloration and partial sloughing of the integuments. Not but that these symptoms occur from other causes. 1. In dropsical persons, there may be anasarcous swelling of the private parts, attended with difficulty of passing urine, and even inflammation and sloughing from distention; 2. an abscess forming near the rectum, and opening into it, and receiving an admixture of fecal matter, I have seen attended by sudden œdematous swelling of the perineum, scrotum, and penis, followed by extensive sloughing of the integuments and cellular membrane, presenting all the external characters of ruptured urethra. 3. I have seen, in consequence of violence done to the testes, sudden and enormous discoloured swelling of the scrotum, perineum, and penis, from the rupture of a vessel, a pint of blood coming away, half clotted, half fluid, on an incision being made. 4. Appearances similar in kind were present in a patient under my care, on whose hip part of a wall had fallen, fracturing the pelvis in several places, at one of which the external iliac vein had been torn, the blood flowing from whence had made its way into the pelvis, perineum, scrotum, and penis.

In a genuine extravasation of urine, from rupture or giving way of the urethra behind a stricture situated near the ligament of Camper, the operation which has been described is peculiarly applicable. Other and additional incisions into the parts swollen with urine are indeed requisite in such a case, in order to give vent to the urine which is diffused through the different parts, and so to lessen the quantity of mortification which must follow; but especially an incision down to the urethra and through the stricture is necessary, in order to allow a catheter to be passed into the bladder, and thus to prevent any *further* infiltration or flow of urine into the cellular membrane, leaving no further demand on the frame than the effort of cleansing the infiltrated scrotum and penis.

II. The same operation is of the utmost service in certain cases of fistula perinæi resulting from stricture.

Cases of stricture complicated with fistula perinæi are of two kinds: the first are those in which the constitution is good, and the stricture in the way of yielding to ordinary means: in such cases let the stricture be enlarged, and when a middling-sized catheter can be passed, let it remain in the urethra, (being changed, of course, frequently, and for a larger one:) not only will the stricture be cured by this means, but the fistula, no longer irritated by the flow of urine through it, will readily close. The second case is that in which the constitution threatens to give way, under the complicated irritation of stricture and fistula with no passage for an instrument through the stricture, or at best a passage through a cartilaginous stricture for a minute catheter only, which would neither serve to dilate the urethra, in such a case, nor prevent the continual flow of the urine through the fistulæ, or the occurrence of fresh infiltrations of urine into the adjacent cellular membrane. To this second case the operation described is perfectly applicable: perform it, and the further flow of the urine through the fistula, or into the cellular membrane, is prevented; the bladder is relieved of the strain of urging the water through narrow and obstructed channels, and the constitution is disembarrassed of all that deranged it.

III. The operation described above is perhaps again applicable to some cases of very narrow stricture of the urethra, unattended with fistula. 1. If such a case were complicated with calculus, and there were reason to suppose that a calculus had made its way into the urethra, and lay behind the stricture, the operation would be strictly applicable. 2. Suppose a case, in which the stricture habitually allowed the water to flow only drop by drop, and that complete retention of urine, not yielding to common remedies, had supervened; it would certainly be a question whether, in such a case, the operation described above should be performed, or the bladder punctured, and slower means of curing the stricture resorted to. 3. Suppose a severe case of stricture, the urine for several weeks having flowed by drops, attended with continual danger of complete retention, or of rupture or ulceration of the urethra, the same question recurs.

In the two last instances, however, the expediency of the operation above described is extremely doubtful. With proper care, either the retention of urine may probably be relieved, or the danger of rupture of the urethra prevented; and by means of armed bougies, or of dilatation, begun by the use of fine catgut bougies, or by the use of catheters perforated at the end, and allowing a lancetted stilette to be thrust forward, (a method revived by Mr. Stafford, which is occasionally advantageous,) the stricture may probably in every instance admit of being cured."

We cannot approve of the tone of despair in which the reporter speaks of cases of fistulæ perinei. He appears to consider them generally as opprobria to surgery, but we know, from our own observation, as well as from the experience of others, that, when they are skilfully managed, they are, most frequently, highly creditable both to the surgeon and his art.

